# Significant predictors of overall survival in patients with hepatocellular carcinoma after surgical resection

**DOI:** 10.1371/journal.pone.0202650

**Published:** 2018-09-04

**Authors:** Chih-Wen Lin, Yaw-Sen Chen, Chih-Che Lin, Po-Huang Lee, Gin-Ho Lo, Chia-Chang Hsu, Pei-Min Hsieh, Kah Wee Koh, Ming-Jong Bair, Chia-Yen Dai, Jee-Fu Huang, Wan-Long Chuang, Yao-Li Chen, Ming-Lung Yu

**Affiliations:** 1 Division of Gastroenterology and Hepatology, E-Da Dachang Hospital, I-Shou University, Kaohsiung, Taiwan; 2 Division of Gastroenterology and Hepatology, Department of Medicine, E-Da Hospital, I-Shou University, Kaohsiung, Taiwan; 3 Health Examination Center, E-Da Hospital, I-Shou University, Kaohsiung, Taiwan; 4 School of Medicine, College of Medicine, I-Shou University, Kaohsiung, Taiwan; 5 Department of Surgery, E-Da Hospital, I-Shou University, Kaohsiung, Taiwan; 6 Department of Surgery, Kaohsiung Chang Gung Memorial Hospital and Chang Gung University College of Medicine, Kaohsiung, Taiwan; 7 Division of Gastroenterology, Department of Internal Medicine, Taitung Mackay Memorial Hospital, and Mackay Medical College, New Taipei City, Taiwan; 8 Hepatobiliary Division, Department of Internal Medicine and Hepatitis Center, Kaohsiung Medical University Hospital, Kaohsiung, Taiwan; 9 College of Biological Science and Technology, National Chiao Tung University, Hsin-Chu, Taiwan; 10 Division of General Surgery, Department of Surgery, Changhua Christian Hospital, Changhua, Taiwan; 11 Institute of Biomedical Sciences, National Sun Yat-sen University, Kaohsiung, Taiwan; University of North Carolina at Chapel Hill School of Medicine, UNITED STATES

## Abstract

**Background:**

The predictive factors of overall survival after hepatectomy for HCC remain controversial and need to be investigated.

**Methods:**

In total, 535 consecutive HCC patients undergoing resection were included and their clinicopathological data and overall survival were recorded. Both the tumor and adjacent non-tumor (ANT) tissues were subjected to immunohistochemistry analysis for the expression of autophagy-related markers.

**Results:**

Death was observed for 219 patients, and the cumulative overall survival rates at 1, 3, 5 and 7 years were 91.0%, 72.3%, 58.8%, and 27.7%, respectively. In the multivariate analysis, mortality was significantly associated with the following: diminished LC3 expression in both the tumor and ANT tissues, in the HCC tissues alone and in the ANT tissues alone (hazard ratio/95% confidence interval: 6.74/2.052–22.19, 6.70/1.321–33.98 and 2.58/1.499–4.915, respectively); recurrent HCC (5.11/3.136–8.342); HBV infection (2.75/1.574–4.784); cirrhosis (1.78/1.059–2.974); and antiviral therapy (0.42/0.250–0.697). The 5-year overall survival rates were 70.2%, 57.3%, 49.6% and 10.7% for patients with positive LC3 expression in both tissue types, in the HCC tissues alone, in the ANT tissues alone, and in neither tissue type, respectively. The 5-year overall survival rates were 56.7%, 47.3%, 51.2% and 38.7% for patients with HBV-related HCC, cirrhosis, no antiviral therapy, and recurrent HCC, respectively, and these rates were significantly lower than those in their counterparts.

**Conclusions:**

Patients with recurrent HCC, HBV-related HCC, cirrhosis, and the absence of antiviral therapy showed significantly lower overall survival rates. Furthermore, LC3 expression in both the tumor and liver microenvironments were significantly predictive of overall survival after resection for HCC.

## Introduction

Hepatocellular carcinoma (HCC) is ranked the fifth most common type of cancer and the third leading cause of cancer-related mortality worldwide [1–3]. HCC occurrence is geographically distributed, with the majority of cases observed in the Asia-Pacific region [4]. In Taiwan, HCC is the second leading cause of cancer-related death [5]. With advancements in early HCC detection techniques, peri-operative management, and surgical procedures, liver resection has become the treatment of choice for patients with operable HCC [6–9]. However, the 5-year recurrence and survival rates after resection for HCC, which are 60% and 50%, respectively, remain unsatisfactory [10, 11]. The detection of HCC during its early stage of development is a crucial factor that affects the prognosis of HCC patients [12]. Hence, the evaluation of predictive factors of overall survival after surgical resection is of clinical relevance and may serve as a promising strategy to improve the postoperative prognoses of HCC patients.

Different predictive factors associated with the prognosis of HCC have been identified and these factors are mostly liver- and tumor-related factors [13, 14]. The etiology of HCC and the severity of cirrhosis are also associated with overall survival in HCC patients [15–17]. The use of postoperative antiviral therapies for viral hepatitis B (HBV)- and hepatitis C (HCV)-related HCC reportedly reduce HCC recurrence and enhance overall survival [18–20]. Different combinations of genes associated with cell proliferation and the tumor microenvironment are also employed as genomic prognostic markers for HCC [21, 22]. The use of these factors to screen and identify patients with a poor postoperative prognosis of HCC may enable more stringent surveillance to prolong their life expectancy. However, the predictive factors for overall survival of HCC patients remain controversial.

Autophagy is a process through which lysosomes are utilized to degrade and recycle damaged organelles for nutrient reuse and energy generation [23]. Autophagy is involved in the physiology and pathogenesis of human liver diseases, including cancer [24, 25]. To date, the role of mammalian autophagy-related markers, namely, Beclin-1 and LC3, as prognostic factors of postoperative HCC has been reported, but the data are conflicting [26–29]. Partial hepatectomy in mice induces autophagy, and such induction is crucial for liver growth, liver regeneration, and survival [30]. In one of our studies, HCC patients with LC3 expression in both the tumor and non-tumor liver microenvironments were significantly protected against post-hepatectomy HCC recurrence [31]. In another study, the accumulation of p62 in the liver microenvironment was significantly correlated with accelerated post-hepatectomy recurrence and reduced disease-free survival [32]. These suggest that autophagy in the liver microenvironment is involved in hepatocarcinogenesis. In addition to regulating the physiological and biological behavior of an organ, normal cellular and non-cellular microenvironmental components are also involved in determining the fates of neighboring tumor cells [33]. The relationship between autophagy in the liver microenvironment and overall survival remains unknown and needs further study. Hence, this study aimed to identify the prognostic factors associated with overall survival in patients who received curative resection for HCC.

## Patients and methods

### Patients and follow-up

A total of 535 consecutive, histologically confirmed HCC patients who underwent curative surgical resection between 2010 and 2014 at E-Da Hospital, Kaohsiung, Taiwan (n = 318) and Changhua Christian Hospital, Changhua, Taiwan (n = 217) were included in this retrospective study. All patients had regular follow-ups every three months after hepatectomy. The follow-up period was defined as the time from the date of the operation to the date of either death or the last follow-up, and the last follow-up was in December 2016. Overall survival was defined as the time from the date of the operation to the date of death or the last follow-up. The patients were divided into those who survived (n = 316) and those who died (n = 219) during the follow-up period after the first hepatectomy. Patients with recurrent HCC were identified based on histological confirmation or at least one typical HCC imaging method according to the recommendations of the American Association for the Study of Liver Disease (AASLD) [34].

The clinicopathological parameters of the patients, including basic demographic data, HCC etiologies, liver function, Child–Pugh score and tumor characteristics, were recorded. The CLIP score includes Child‐Pugh stage, tumor morphology and extension, serum AFP levels, and portal vein thrombosis. Among the 535 patients, 123 (23%) patients had received liver resection consisting of either 3–4 segmentectomy (11.8%) or > 4 segmentectomy (11.2%) and 412 (77.0%) patients had received minor liver resection (≤ 2 segmentectomy). The definite of cirrhosis is the fibrotic stage 4 of non-tumor part from the resection liver by histology and non-cirrhosis is the fibrotic stage 0–3. The resected HCC tumor tissues, together with the paired adjacent non-tumor (ANT) tissues, which were 0.5–5 cm from the negative operative margin, were collected and stored in 4% paraformaldehyde until use. Antiviral therapy was defined as patients with HBV, HCV, or HBV/HCV infection receiving treatment according to the Taiwan Association for the Study of the Liver. A total of 65.2% (163/250) of the patients with HBV infection were treated with nucleoside/nucleotide analogs. A total of 37.5% (57/152) of the patients with HCV infection were treated with pegylated interferon with ribavirin or direct-acting antiviral agents and achieved sustained virologic responses. A total of 85.7% (18/21) of patients with HBV/HCV co-infection were treated with nucleoside/nucleotide analogs, pegylated interferon with ribavirin or direct-acting antiviral agents. This study was approved by the Institutional Review Board of the E-DA Hospital (EMRP32100N) and the Institution of Reviewer Board of Changhua Christian Hospital (091107). The experiments were conducted in accordance with the guidelines of the International Conference on Harmonization for Good Clinical Practice. All participants were adult and provided written informed consent.

### Tissue microarray construction

Tissue microarrays were constructed according to the manufacturer’s instructions (Array Biotechnology Co., Taiwan). Briefly, all HCC specimens were stained with hematoxylin and eosin (H&E), and representative areas that were free from necrotic and hemorrhagic materials were marked in the paraffin blocks. Two cylindrical tissue cores (1.6-mm diameter) were removed from the donor blocks and transferred to the recipient paraffin blocks, and their planar array positions were noted. Each contained approximately 96 cylinders, and the final tissue microarrays consisted of 535 HCC tissue samples along with paired ANT tissues. The 4-μm-thick consecutive sections obtained from the array blocks were placed on adhesion microscope slides for immunohistochemistry (IHC).

### Immunohistochemical staining and scoring

The 4-μm tissue sections were stained using the HRP (DAB) detection system according to the manufacturer’s instructions with slight modifications. The primary polyclonal antibodies used were anti-LC3 (NB100-2220, Novus Biologicals, Littleton, CO, USA), anti-Beclin-1 (ab51031, Abcam, Cambridge, UK), and anti-p62 (H00008878-M01, Abnova, Taipei, Taiwan). The expression of autophagy-related proteins (LC3, Beclin-1, and p62) was quantitated using the semi-quantitative immunoreactive scoring (IRS) system as described previously [35], and the expression was defined as either positive (IRS ≥ 2) or negative (IRS <2) based on the products of the intensity and percentage scores. All slides were evaluated independently by two investigators in a blinded manner. Cases with discrepancies were discussed until a consensus was reached.

### Data analysis and statistics

All statistical analyses were performed using SPSS ver. 18.0 (SPSS, Chicago, IL, USA). The associations between markers and clinical characteristics were evaluated using Pearson’s χ2 test and Fisher’s exact test, where appropriate. Group means (mean±standard deviation) were compared using analysis of variance and Student’s t-test, where appropriate. Correlation coefficients between each marker was determined using Spearman’s correlation analysis. To evaluate whether the variables selected in the univariate analysis were independent factors associated with overall survival, multivariate analyses were performed using a Cox’s proportional hazard regression model, and the results were reported as the hazard ratio (HR) with 95% confidence intervals (CIs). Variables including sex, age, Platelet count, AFP, Hepatitis etiology, liver cirrhosis, antiviral therapy, tumor recurrence, marcovascular invasion, microvascular invasion, tumor size, BCLC stage, CLIP score, and LC3 in tumor/ANT tissues were entered into a multivariate analysis. Kaplan-Meier survival curves were plotted and compared using the log-rank test to examine the differences in survival with respect to different factors. All statistical analyses were two-sided, and a *p*-value<0.05 was considered significant.

## Results

### Baseline demographic and clinicopathological data

Table 1 shows the demographic data and clinicopathological factors of the 535 patients. Two hundred and nineteen (40.9%) patients died. The study group predominantly comprised male patients (73.1%), and the mean age was 63 years. Pre-existing diseases, namely, hypertension and diabetes mellitus, were observed in 18.9% and 11.0% of the patients, respectively. Regarding HCC etiologies, 28.4%, 46.7%, 3.9% and 20.9% of the patients had HCV-, HBV-, HBV/HCV-co-infection- and non-viral-related HCC, respectively. Of the 423 patients with viral hepatitis, 56.3% had received antiviral therapy. Cirrhosis was observed in 32.3% of the patients. Regarding tumor histology and pathological stage, 9.5% of the patients had an Edmondson–Steiner grade of I-II, and most patients had TNM stage I-II (83.6%), BCLC stage 0-A (63.9%) disease, and CLIP score 0–1 (80.0%). HCC recurred in 45.8% of the patients, with 116 patients having recurrences within 2 years post-hepatectomy (early recurrence), and 129 patients having recurrences at least 2 years after hepatectomy (late recurrence). Extra- and intra-hepatic recurrences were observed in 17.1% and 82.9% of the patients, respectively. The examination of the autophagy-related markers revealed the following: 91.6% of the HCC tissues and 59.8% of the ANT tissues were positive for LC3; 86.7% of the HCC tissues and 34.8% of the ANT tissues were positive for Beclin-1; and 81.1% of the HCC tissues and 8.4% of the ANT tissues were positive for p62.

**Table 1:** Basic demographic data and univariate analysis of overall survival of hepatocellular carcinoma patients who underwent curative resection.

Characteristics	All patients (n = 535)	Non-mortality (n = 316)	Mortality (n = 219)	*p*-value
Gender				
*Female*	144 (26.9)	74 (23.4)	70 (32.0)	**0.030**
*Male*	391 (73.1)	242 (76.6)	149 (68.0)	
Age (years)	63.1±11.5	62.5±12.1	64.0±10.5	0.141
HTN	101 (18.9)	55 (17.4)	46 (21.0)	0.368
DM	59 (11.0)	39 (12.3)	20 (9.1)	0.264
Alcohol	129 (24.9)	73 (23.1)	56 (25.6)	0.538
Smoking	152 (28.4)	99 (31.3)	53 (24.2)	0.080
HCC etiology				
*HCV*	152 (28.4)	102 (32.3)	50 (22.8)	**0.015**
*HBV*	250 (46.7)	141 (44.6)	109 (49.8)	
*Non HBVHCV*	112 (20.9)	57 (18.0)	55 (25.1)	
*HBV+HCV*	21 (3.9)	16 (5.1)	5 (2.3)	
AST (IU/L)	55±38	53±36	57±40	0.225
ALT (IU/L)	50± 39	50±35	51±43	0.774
Total Bilirubin (mg/dl)	0.79±0.34	0.80±0.36	0.79±0.31	0.854
Albumin (g/dl)	3.9±0.4	3.9±0.5	3.8±0.4	0.445
Creatinine	1.0±0.7	1.0±0.7	1.1±0.9	0.540
Platelet count (x10^3^/ml)	175±71	169±72	184±69	**0.013**
INR	1.07±0.10	1.09±0.14	1.07±0.12	0.315
AFP (ng/dl)				
*< 400*	438 (81.9)	260 (82.3)	178 (81.3)	0.768
*≥ 400*	97 (18.1)	56 (17.7)	41 (18.7)	
ICG (%)	8.3±5.3	8.4±5.4	8.2±5.2	0.759
Liver cirrhosis				
*Negative*	362 (67.7)	227 (71.8)	135 (61.6)	**0.013**
*Positive*	173 (32.3)	89 (28.2)	84 (38.4)	
Antiviral therapy				
*Negative*	185 (43.7)	98 (37.8)	87 (53.0)	**0.002**
*Positive*	238 (56.3)	161 (62.2)	77 (47.0)	
Operative methods				
*Minor LR*	412 (77.0)	250 (79.1)	162 (74.0)	0.360
*Major LR*	123 (23.0)	66 (20.9)	57 (26.0)	
Operative margin (>1 cm)				
*Negative*	150 (28.0)	95 (30.1)	55 (25.1)	0.240
*Positive*	385 (72.0)	221 (69.9)	164 (74.9)	
Edmondson-Steiner Grades				
*I-II*	51 (9.5)	28 (8.9)	23 (10.5)	0.551
*III-IV*	484 (90.5)	288 (91.1)	196 (89.5)	
Macrovascular invasion				
*Negative*	424 (79.3)	261 (82.6)	163 (74.4)	**0.022**
*Positive*	111 (20.7)	55 (17.4)	56 (25.6)	
Microvascular invasion				
*Negative*	289 (54.0)	185 (58.5)	104 (47.5)	**0.012**
*Positive*	246 (46.0)	131 (41.5)	115 (52.5)	
Tumor number				
*Single*	438 (81.9)	250 (79.1)	188 (85.8)	0.052
*Multiple*	97 (18.1)	66 (20.9)	31 (14.2)	
Tumor size				
*< 5 cm*	352 (65.8)	220 (69.6)	132 (60.3)	**0.026**
*≥5 cm*	183 (34.2)	96 (30.4)	87 (39.7)	
TNM stage				
*I-II*	447 (83.6)	262 (82.9)	185 (84.5)	0.722
*III-IV*	88 (16.4)	54 (17.1)	34 (15.5)	
BCLC stage				
*0-A*	342 (63.9)	214 (67.7)	128 (58.4)	**0.035**
*B-C*	193 (36.1)	102 (32.3)	91 (41.6)	
CLIP score				
*0–1*	428 (80.0)	254 (80.4)	174 (79.5)	0.792
*2–5*	107 (20.0)	62 (19.6)	45 (20.5)	
HCC recurrence status				
*Negative*	290 (54.2)	226 (71.5)	64 (29.2)	**< .0001**
*Positive*	245 (45.8)	90 (28.5)	155 (70.8)	
LC3 in tumor tissues				
*Negative*	45 (8.4)	9 (2.8)	36 (16.4)	**< .0001**
*Positive*	490 (91.6)	307 (97.2)	183 (83.6)	
Beclin-1 in tumor tissues				
*Negative*	71 (13.3)	47 (14.9)	24 (11.0)	0.198
*Positive*	464 (86.7)	269 (85.1)	195 (89.0)	
p62 in tumor tissues				
*Negative*	101 (18.9)	56 (17.7)	45 (20.5)	0.433
*Positive*	434 (81.1)	260 (82.3)	174 (79.5)	
LC3 in ANT tissues				
*Negative*	215 (40.2)	98 (31.0)	117 (53.4)	**< .0001**
*Positive*	320 (59.8)	218 (69.0)	102 (46.6)	
Beclin-1 in ANT tissues				
*Negative*	349 (65.2)	216 (68.4)	133 (60.7)	0.079
*Positive*	186 (34.8)	100 (31.6)	86 (39.3)	
p62 in ANT tissues				
*Negative*	490 (91.6)	287 (90.8)	203 (92.7)	0.527
*Positive*	45 (8.4)	29 (9.2)	16 (7.3)	

Data shown as mean ± standard deviation or number (%). HTN: Hypertension; DM: Diabetes Mellitus; HBV: Hepatitis B virus; HCV: Hepatitis C virus; AST: aspartate aminotransferase; ALT: alanine aminotransferase; INR: International normalized ratio; AFP: Alpha-fetoprotein; ICG: Indocyanine green; Minor liver resection: ≤ 2 segmentectomy; Major liver resection: ≥ 3 segmentectomy; BCLC stage: Barcelona clinic liver cancer; ANT part, adjacent non-tumor part.

### Factors related to overall survival in patients who underwent curative hepatectomy for HCC

**Fig 1:**
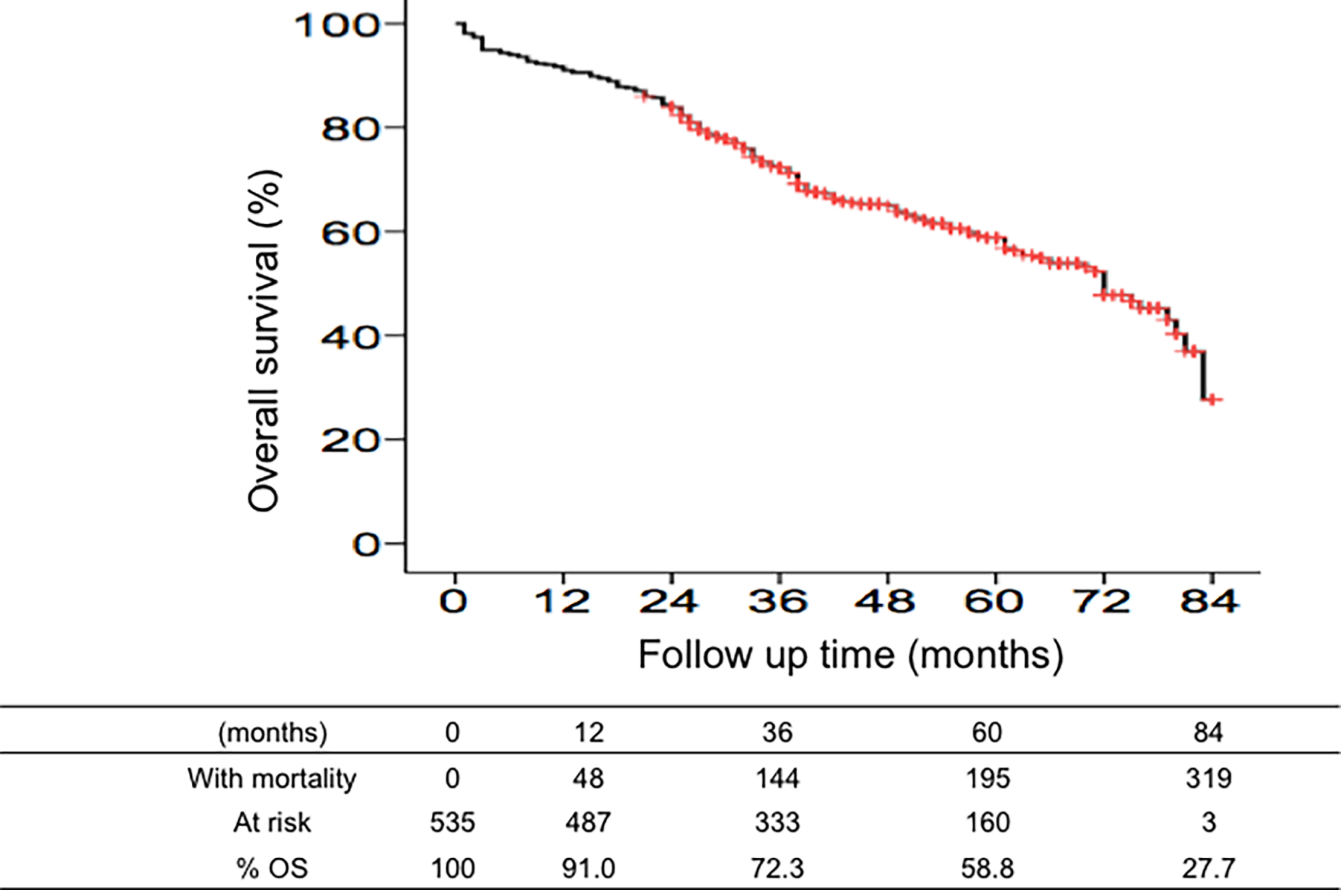
Cumulative incidence of overall survival calculated using Kaplan-Meier survival analysis.

During the median follow-up of 42 months (range, 1 to 84 months), 59.1% of the patients remained alive, and the cumulative incidences of overall survival at 1, 3, 5 and 7 years was 91.0%, 72.3%, 58.8%, and 27.7% (Fig 1). In univariate analysis, sex, age, pre-existing disease, liver function, AFP, operative method, operative margins, tumor number, CLIP score were not significantly different between the surviving patients and those who died (Table 1). Conversely, the following factors were significantly associated with higher overall survival: HCC etiology such as HCV infection, platelet count, absence of cirrhosis, patients receiving antiviral therapy for viral hepatitis, absence of macrovascular and microvascular invasions, tumor size <5 cm, BCLC stage 0-A, absence of HCC recurrence, and the presence of LC3 expression in HCC tissues or ANT tissues.

In multivariate analysis, the Cox proportional hazard model showed that patients lacking LC3 expression in both the HCC and ANT tissues had the highest mortality (-/-; HR: 6.74; 95% CI: 2.052–22.19, *p*<0.0001), followed by those lacking LC3 expression in the HCC tissues alone (-/+; HR: 6.70; 95% CI: 1.321–33.98, *p* = 0.022), those with recurrent HCC (HR: 5.11; 95% CI: 3.136–8.342, *p*<0.0001), those with HBV-related HCC (HR: 2.75; 95% CI: 1.574–4.784, *p*<0.0001), those lacking LC3 expression in the ANT tissues alone (+/-; HR: 2.58; 95% CI: 1.499–4.915, *p =* 0.002), and those with cirrhosis (HR: 1.78; CI: 1.059–2.974, *p* = 0.029) (Table 2). The mortality was also significantly reduced in patients receiving antiviral therapy (HR: 0.42; CI: 0.250–0.697, *p* = 0.001).

**Table 2:** Multivariate analyses of factors associated with mortality of hepatocellular carcinoma patients who underwent curative resection.

Variables	Hazard ratio	95% CI	*p*-value
Sex			
*Female*	1		
*Male*	0.60	0.349–1.026	0.062
Age (years)			
*< 65*	1		
*≥ 65*	1.09	0.792–1.526	0.572
Platelet count (x10^3^/ml)			
*< 100*	1		
*≥ 100*	1.38	0.691–2.38	0.058
AFP (ng/dl)			
*< 400*	1		
*≥ 400*	1.09	0.776–1.531	0.620
Hepatitis etiology			
*HCV*	1		
*HBV*	2.75	1.574–4.784	**<0.0001**
*Non HBVHCV*	1.38	0.363–5.213	0.639
*HBV+HCV*	0.93	0.282–3.053	0.903
Liver cirrhosis			
*Negative*	1		
*Positive*	1.78	1.059–2.974	**0.029**
Antiviral therapy			
*Negative*	1		
*Positive*	0.42	0.250–0.697	**0.001**
Tumor recurrence			
*Negative*	1		
*Positive*	5.11	3.136–8.342	**<0.0001**
Macrovascular invasion			
*Negative*	1		
*Positive*	1.05	0.459–2.407	0.906
Microvascular invasion			
*Negative*	1		
*Positive*	2.17	0.925–5.092	0.075
Tumor size			
*< 5 cm*	1		
*≥5 cm*	1.61	0.724–3.594	0.242
BCLC stage			
*0-A*	1		
*B-C*	0.50	0.184–1.343	0.168
CLIP score			
*0–1*	1		
*2–5*	1.09	0.792–1.526	0.572
LC3 in tumor/ANT tissues			
*+/+*	1		
*+/-*	2.58	1.499–4.915	**0.002**
*-/+*	6.70	1.321–33.98	**0.022**
*-/-*	6.74	2.052–22.19	**<0.0001**

ANT, adjacent non-tumor; BCLC stage: Barcelona clinic liver cancer

**Fig 2:**
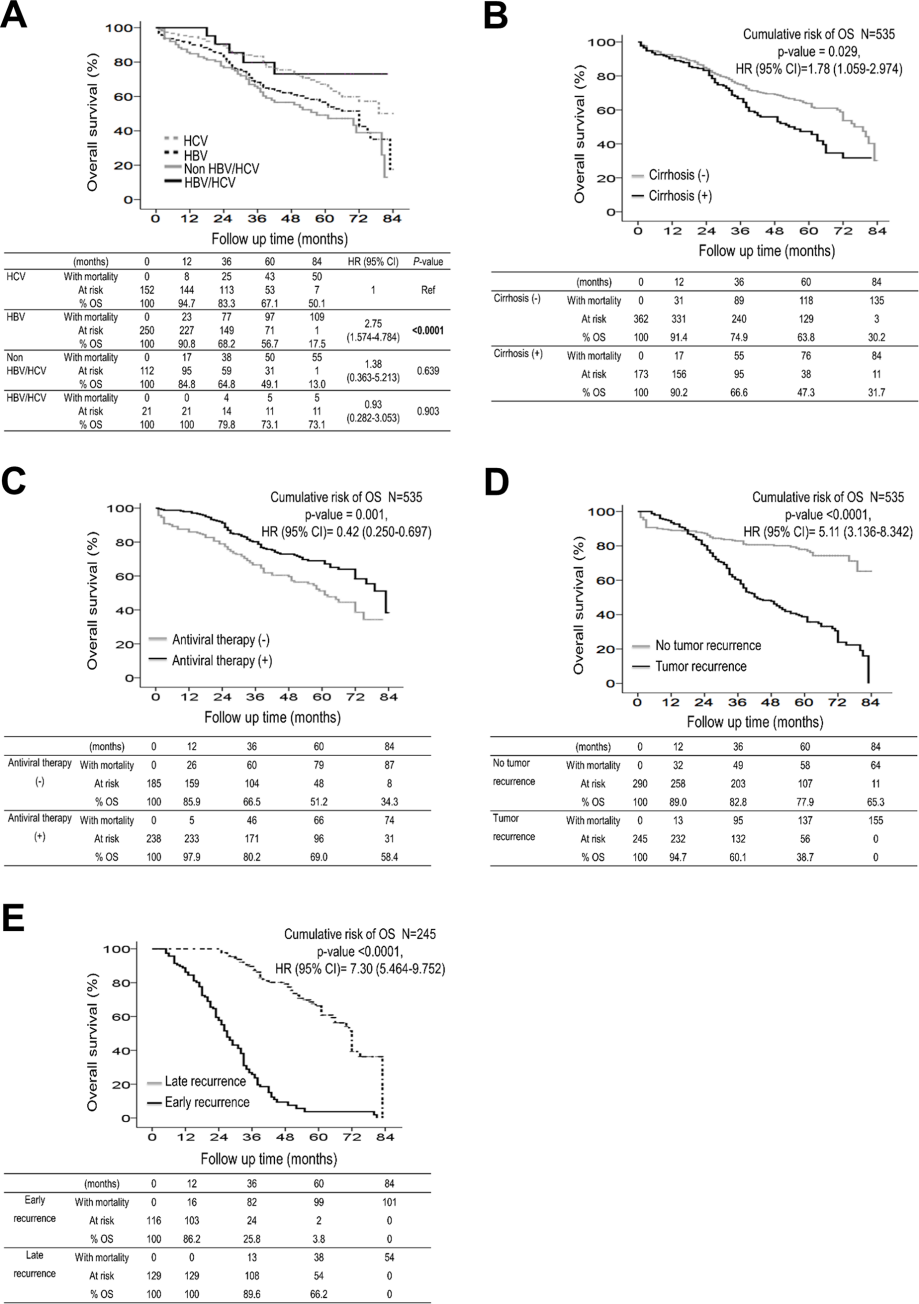
Cumulative incidence of overall survival with respect to various clinicopathological factors. Patients with HCV-related HCC (A), the absence of liver cirrhosis (B), antiviral therapy for hepatitis viral infection (C), the absence of recurrent HCC (D), and late recurrence (E) had significantly higher overall survival rates than their counterparts.

**Fig 3:**
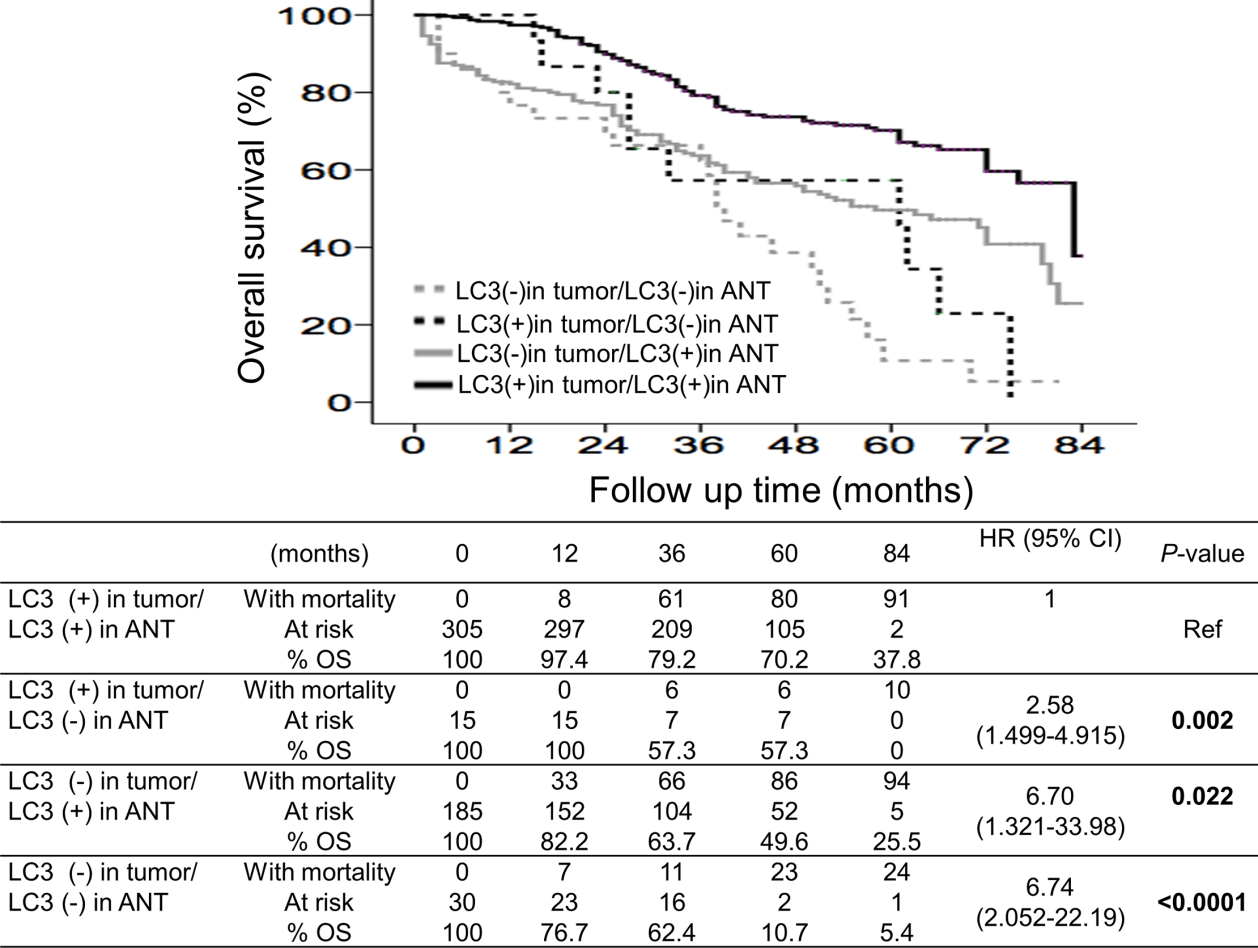
Cumulative incidence of overall survival with respect to LC3 expression in both the tumor and adjacent non-tumor tissues. Patients with LC3 expression in both the HCC and ANT tissues (+/+) had a significantly higher overall survival rate than patients lacking LC3 expression in the ANT tissues (+/-), in the HCC tissues (-/+) or both tissue types (-/-).

Kaplan-Meier survival analysis revealed that patients with HCV-related HCC had significantly higher overall survival than their counterparts (Fig 2A, log-rank test), and their 1-, 3-, 5- and 7-year overall survival rates were 94.7%, 83.3%, 67.1% and 50.1%, respectively. Patients without cirrhosis had significantly higher overall survival rates than their counterparts, and their 1-, 3-, 5- and 7-year overall survival rates were 91.4%, 74.9%, 63.8% and 30.2%, respectively (Fig 2B). For patients who had received antiviral therapy for hepatitis viral infection, their overall survival rates were significantly higher than their counterparts, having 1-, 3-, 5- and 7-year overall survival rates of 97.9%, 80.2%, 69.0% and 58.4%, respectively (Fig 2C). For patients without recurrent HCC, their overall survival rates were significantly higher than their counterparts, having a 1-, 3-, 5- and 7-year overall survival rates of 89.0%, 82.8%, 77.9% and 65.3%, respectively (Fig 2D). A comparison between patients with early and late recurrences revealed that those with late recurrences had significantly higher overall survival rates (*p*<0.0001, Fig 2E). However, all patients with recurrent HCC eventually died during the follow-up period. Patients with positive LC3 expression in both the HCC and ANT tissues (+/+) had the best survival rates, and their 1-, 3-, 5- and 7-year overall survival rates were 97.4%, 79.2%, 70.2% and 37.8%, respectively (Fig 3). The overall survival rates were significantly lower for patients lacking LC3 expression in both tissues (-/-, *p*<0.0001), in the HCC tissues alone (-/+, *p*<0.022) and in the ANT tissues alone (+/-, *p* = 0.002).

## Discussion

In the current study, 535 consecutive HCC patients who underwent curative hepatectomy at two hospitals were examined to identify factors affecting overall survival. Our results demonstrated that the presence of autophagy-related marker LC3 in both the tumor and non-tumor liver microenvironments is significantly associated with higher overall survival. This finding suggests that the LC3 expression in both microenvironments has a protective role against mortality and that the measurement of LC3 expression in both tissues may serve as an excellent predictor of overall survival for patients with curative hepatectomy for HCC. In addition, HCC etiology, cirrhosis, antiviral therapy and recurrent HCC are also risk factors that affect the prognosis of HCC after postoperative hepatectomy.

The overall survival after curative hepatectomy is often impaired by the high rates of recurrence and tumor-related death observed [34]. HCC recurrence has a poorer survival outcome [36, 37]. In two separate studies, the 5-year overall survival rates of postoperative HCC patients with recurrent HCC were 30.9% and 38%, which were significantly poorer than those without recurrent HCC, having 5-year overall survival rates of 72.9% and 85%. In our study, the 5-year overall survival rates of patients with and without recurrent HCC were 38.7% and 77.9%, respectively, which were similar to the observations made in the two studies [38, 39]. Although patients with early recurrences had a poorer 5-year overall survival rate than those with late recurrences (3.8% vs 66.2%), all patients with recurrent HCC eventually died, suggesting that HCC recurrence has a major impact on the postoperative prognosis of HCC patients.

The impairment of liver function caused by cirrhosis may restrict the treatment modalities available to treat HCC, and therefore, patients with severe cirrhosis are seldom recommended for hepatectomy. Cirrhosis is also one factor that affects the prognosis of postoperative HCC [40, 41]. Several studies have revealed that the 5-year overall survival rates of postoperative HCC patients with cirrhosis range from 42.2–50% and are significantly poorer than those without cirrhosis, having 5-year overall survival rates ranging from 51–73% [40, 41]. The degree of cirrhotic severity is reportedly associated with postoperative overall survival, and the overall survival rate of patients with increased liver fibrosis or cirrhosis severity is worsened [41–43]. In our study, the 5-year overall survival rate of the patients with cirrhosis was significantly poorer than those without cirrhosis (47.3% vs. 63.8%), which was similar to these studies. This finding further supports the impact of cirrhosis on postoperative overall survival. Of the 245 patients who had experienced recurrent HCC, cirrhosis was associated with a higher risk of developing recurrent HCC (HR: 1.59, p<0.017; Under Review). Given that cirrhotic severity is associated with recurrent HCC and that cirrhosis is associated with poorer overall survival (HR: 5.11, p<0.0001), the data further demonstrate that the presence of coexisting cirrhosis is associated with a higher risk of recurrent HCC and poorer overall survival rates in HCC patients with curative hepatectomy.

The use of antiviral therapies to suppress HBV viral replication and achieve SVR for HCV infection is significantly associated with prolonged recurrence-free survival and overall survival in patients with curative hepatectomy for HBV- and HCV-related HCC [18–20, 44, 45]. In one study, the 5-year overall survival rates of postoperative HBV-related HCC patients with and without antiviral therapy were 66.7% and 56%, respectively [18]. In another study, the 4-year overall survival rates of both non-randomized and randomized cohorts were 59.6% and 86.4%, respectively, for HBV-related HCC patients with antiviral therapy and 46.6% and 47.4%, respectively, for patients without antiviral therapy [19]. In both studies, the enhanced survival rates observed for patients receiving antiviral therapy were associated with a better liver function reserve during the time of recurrence, hence allowing subsequent curative treatment to be performed [18, 19]. In another study conducted in Taiwan, the 6-year overall survival rates of HBV-related HCC patients with and without antiviral therapy after postoperative hepatectomy were 71% and 57.6%, respectively [46]. For HCV-related HCC, the 5-year overall survival rate of patients receiving antiviral therapy for HCV infection after hepatectomy was significantly higher than the survival rate of the patients without antiviral therapy (91.7% vs. 50.6%) [47]. In another study comparing the overall survival rates of postoperative HCV-related HCC patients with high vs. low HCV viral loads, patients with low viral loads denoting less severe activity of hepatitis had a significantly better overall survival rate than those with high viral loads (76.6% vs. 57.7%) [44]. In our study, although patients receiving antiviral therapy were not separated based on HCV and HBV infection, the 5-year overall survival rate of patients receiving antiviral therapy was significantly better than those without antiviral therapy (69.0% vs. 51.2%, p<0.0001). These data suggest that the treatment for active hepatitis is crucial in the management of HCC.

A previous study showed that the expression of LC3 in HCC tissues is significantly associated with longer overall survival and a longer time to recurrence in postoperative patients [27]. However, another study showed that high LC3 expression in HCC tissues is associated with poor overall survival [48]. In our study, patients with LC3 expression in both the tumor and liver microenvironments (+/+) had a better clinical outcome than those lacking LC3 expression in both tissue types (-/-), in the HCC tissue alone (-/+), and in the liver microenvironment alone (+/-). Given that patients lacking LC3 in the HCC tissues (both -/- and -/+) had an almost similar risk of mortality (HR 6.74 vs. 6.70, respectively), the presence of LC3 in the HCC tissues may have a high impact on protection against mortality, and LC3 expression in the HCC tissues may be important for enhancing overall survival. Notably, patients lacking LC3 in the ANT tissues alone (+/-) were at high risk of mortality (HR 2.58), and all of these patients eventually died at the end of the follow-up period. This observation suggests that the presence of LC3 in the ANT tissues is equally important for protecting against mortality and support the use of LC3 expression in the tumor and ANT tissues as a prognostic factor for overall survival in patients with curative hepatectomy for HCC. Furthermore, LC3 expression is not significantly associated with clinicalpathological variables such as marcovascular invasion, microvascular invasion, tumor differentiation, tumor size, tumor number, TNM stage, BCLC stage and CLIP score. LC3 expression is significantly associated with tumor recurrence. Our previous study showed that LC3 expression is significantly associated with early recurrence, late recurrence, and all recurrence, respectively [31]. Tumor recurrence is significantly associated with overall survival. It is possible that LC3 expression is significantly associated with overall survival because of tumor recurrence.

The relationship between LC3 expression in the non-tumor microenvironment and overall survival has not been discussed in the literature. We have previously reported that a lack of LC3 expression in ANT tissues is associated with immediate death post-hepatectomy [35]. Here, we show that a lack of LC3 expression in both the tumor and non-tumor liver microenvironments is strongly associated with the poor prognosis of patients with curative hepatectomy for HCC. The presence of LC3 expression in both tissue types has protective effects against mortality, suggesting the importance and involvement of autophagy in both tissue types in affecting the overall survival of HCC after hepatectomy. In addition, we have also found that LC3 expression in the tumor and liver microenvironments is significantly associated with HCC recurrence [31]. Overall, we demonstrate that the LC3 expression in the non-tumor liver microenvironment has a significant effect on the clinical prognosis, including immediate mortality, overall survival, and HCC recurrence, in patients with curative resection for HCC. This observation suggests that autophagy in the non-tumor liver microenvironment plays roles in the postoperative prognosis of HCC and that the inclusion of IHC examination of ANT tissues for LC3 expression during hepatectomy can provide additional information for critical surveillance, clinical prognosis, and supportive therapy.

In summary, the absence of LC3 expression in both the tumor and non-tumor liver microenvironments is significantly associated with poor overall survival in patients who undergo curative hepatectomy for HCC. Factors such as HCC etiology, cirrhosis, antiviral therapy for hepatitis viral infection, and recurrent HCC are also associated with a poor prognosis of HCC. In addition to the tumor microenvironment, the assessment of autophagy function in the non-tumor liver microenvironment is equally important for predicting overall survival. The analysis of LC3 expression in tumor and ANT tissues, in conjunction with an assessment of HCC recurrence status, HCC etiology, the presence of cirrhosis and antiviral therapy status, can aid in identifying patients at risk of mortality after curative resection. Our results suggest that autophagy plays an important role in the prognosis of patients with curative hepatectomy for HCC and that LC3 may serve as a marker for predicting overall survival and as a potential therapeutic target for enhancing the life expectancy of HCC patients.

## Supporting information

S1 Dataset (XLSX)
